# Clinical predictors of coronary artery bypass graft surgery in patients hospitalized for Non-ST acute coronary syndrome - Buenos Aires I and ReSCAR22 registries

**DOI:** 10.47487/apcyccv.v5i1.333.

**Published:** 2024-03-19

**Authors:** Julián M. Feder, Alan R. Sigal, Leonardo A. Seoane, Mirza Rivero, Gonzalo Perez, Ezequiel J. Zaidel, Fabricio G. Procopio, Diego Conde, Juan P. Costabel

**Affiliations:** 1 Instituto Cardiovascular de Buenos Aires, Buenos Aires, Argentina. Instituto Cardiovascular de Buenos Aires Buenos Aires Argentina; 2 CEMIC, Buenos Aires, Argentina. CEMIC Buenos Aires Argentina; 3 Clínica Olivos, Buenos Aires, Argentina. Clínica Olivos Buenos Aires Argentina; 4 Sanatorio Güemes, Buenos Aires, Argentina. Sanatorio Güemes Buenos Aires Argentina; 5 Hospital Universitario Fundación Favaloro, Buenos Aires, Argentina. Hospital Universitario Fundación Favaloro Buenos Aires Argentina

**Keywords:** Myocardial Revascularization, Coronary Artery Disease, Acute Coronary Syndrome, Argentina

## Abstract

**Objectives.:**

To identify predictors of coronary artery bypass graft surgery (CABG) requirement as a revascularization method in in real-world non-ST-segment elevation acute coronary syndrome (NSTE-ACS) patients.

**Materials and methods:**

. An individual pre-specified analysis of patients with NSTE-ACS was performed from two prospective Argentine registries between 2017 and 2022. We analyzed the difference in baseline characteristics between patients who required CABG and those who did not require this intervention. Then, a logistic regression analysis was performed to determine independent predictors in patients who received CABG as a method of revascularization.

**Results.:**

A total of 1848 patients with a median age of 54.8 (interquartile range [IQR]: 53.7-56) years and an ejection fraction of 42.1% (IQR: 41.2-43.1) were included. A total of 233 patients required CABG (12.6%). Baseline characteristics between the two groups were similar, except in patients requiring CABG, who were younger (51.5 *vs.* 55.7 years; p=0.010), more frequently diabetic (38.2% *vs.* 25.7%; p=0.001) and male (90.1% *vs.* 73.7%; p=0.001). In addition, they had, to a lesser extent, previous cardiac surgery (2.1% *vs.* 11.2%; p=0.011). After multivariable analysis, the following were independently associated with CABG: age (Odds Ratio [OR]: 0.99, 95% confidence interval [CI]: 0.98-0.99; p=0.008), male sex (OR: 3.08, 95% CI: 1.87-5.1; p=0.001), history of previous CABG (OR: 0.14, 95% CI: 0.05-0.30; p=0.001) and diabetes (OR: 1.84, 95% CI: 1.31- 2.57; p=0.001).

**Conclusions.:**

In this analysis of two NSTEACS registries, younger age, male sex, a diagnosis of diabetes and the absence of previous surgery were independent predictors of the requirement for inpatient CABG.

## Introduction

In patients admitted to a coronary care unit with a diagnosis of non-ST-segment elevation acute coronary syndrome (NSTE-ACS), the recommendation according to clinical practice guidelines is to perform revascularization as completely as possible, considering the difficulty in many cases of identifying the responsible lesions ^1^^,^^2^. The decision on which type of revascularization strategy to use is usually guided by the anatomical complexity and clinical characteristics of the patient, with percutaneous coronary intervention (PCI) being more common. Generally, the literature shows that between 5 and 15% of NSTE-ACS patients require coronary artery bypass graft surgery (CABG) ^2^^-^^8^. This type of strategy has implications in terms of antiplatelet therapies and length of hospital stay. Therefore, the primary objective of this study is to conduct a retrospective analysis on a population-based sample from our region, aiming to evaluate which clinical variables were statistically associated with the choice of CABG over PCI in high-complexity and high-volume centers in the Autonomous City of Buenos Aires (ACBA) and the Province of Buenos Aires (PBA).

## Materials and methods

### Design and study population

A pre-specified analysis was conducted on patients included in two multicenter acute coronary syndrome registries in Argentina: the Buenos Aires I registry and the ReSCAR22 registry. Both registries enrolled patients from high-volume and high-complexity centers, mainly from the PBA and ACBA, prospectively and consecutively. These registries were designed and conducted by the Emergency and Critical Cardiology Council of the Argentine Society of Cardiology.

The Buenos Aires I registry was a prospective observational study conducted from December 2017 to July 2018. This registry included 1100 consecutive patients with NSTE-ACS from 21 centers ^9^. ReSCAR was a multicenter prospective observational registry that included patients from January to August 2022 ^10^. This registry included 984 patients with acute coronary syndrome (ACS) with or without ST-segment elevation from 15 centers. For the present analysis, only baseline data from patients with complete data were used, and patients from both registries who were diagnosed with NSTE-ACS according to the fourth universal definition of myocardial infarction were included ^11^.

### Variables

The variables used in this study were selected by the central registry committee:

#### Personal history

Cardiovascular risk factors, clinical history, and relevant comorbidities were obtained through medical history at the time of hospital admission. Personal history of hypertension, diabetes mellitus, dyslipidemia, smoking (current or past), sedentary lifestyle, family history of premature cardiovascular disease, chronic obstructive pulmonary disease (COPD), and chronic kidney disease (CKD) were evaluated. Additionally, information on cardiovascular history, such as myocardial infarction (MI), PCI, CABG, stable angina (SA), stroke, transient ischemic attack (TIA), atrial fibrillation (AF), peripheral vascular disease (PVD), and previous bleeding events, were obtained.

#### Characteristics of ACS

The data related to ACS were obtained from the patient’s medical history, according to:

- a) Type of ACS: unstable angina, NSTE-ACS, ST-segment elevation acute coronary syndrome (STE-ACS), type 2 MI, myocardial infarction with non-obstructive coronary arteries (MINOCA), myocarditis, or Takotsubo syndrome.
- b) Killip and Kimball (KK) classification at the time of hospital admission.
- c) Electrocardiographic changes: ST-segment elevation, ST-segment depression, T-wave changes, Q waves associated with ST-segment or T-wave abnormalities, left bundle branch block, pacemaker rhythm, or no evidence of acute ischemic changes.
- d) Initial strategy used (invasive or conservative) and time to coronary angiography (CAG).
- e) Treatment instituted: acetylsalicylic acid (aspirin), P2Y12 receptor inhibitor (P2Y12i) used and time of prescription, as well as anticoagulant treatment and the chosen agent.
- f) CAG result, type of lesions, number of vessels involved, and type of stent used.
- g) Ischemic complications: 1) recurrent angina, refractory angina, post-MI angina; 2) re-MI; 3) intra-stent thrombosis; 4) need for CABG; and 5) stroke or TIA.
- h) Electrical complications: AF, ventricular tachycardia or fibrillation, and high-grade atrioventricular block.
- i) Mechanical complications: ventricular septal defect, acute mitral regurgitation, and cardiac rupture.
- j) Requirement for mechanical ventilation or ventricular assistance.
- k) Other complications: contrast-induced nephropathy, acute kidney injury (AKI), and heart failure.
- l) In-hospital bleeding according to the BARC classification 1-5.
- m) In-hospital death.
- n) Treatment at the time of hospital discharge: antiplatelet therapy (aspirin, clopidogrel, prasugrel, or ticagrelor), oral anticoagulation, beta-blockers (BB), angiotensin-converting enzyme inhibitors (ACEIs), angiotensin II receptor blockers (ARBs), statins, ezetimibe, aldosterone antagonists, nitrates, trimetazidine, and calcium channel blockers.
- o) Total length of hospital stay.
- p) Calculation of the GRACE (The Global Registry of Acute Coronary Events) and CRUSADE (Can Rapid Risk Stratification of Unstable Angina Patients Suppress Adverse Outcomes With Early Implementation of the ACC/AHA Guidelines) scores. Both scores were calculated based on the variables suggested by their creators. CRUSADE score (https://qxmd.com/calculate/calculator_ 53/crusade-score-for-post-mi-bleeding-risk) and GRACE score (https://www.outcomes-umassmed.org/grace/acs_risk2/index.html).

### Data analysis

Continuous variables were expressed as mean and standard deviation (SD) or median and interquartile range (IQR), depending on the distribution characteristics. Normality of distribution was assessed using the Kolmogorov-Smirnov or Shapiro-Wilk test, as appropriate. Categorical variables were analyzed using the chi-square test or Fisher’s exact test, while numerical variables were analyzed using Student’s t-test or the Mann-Whitney U test, depending on their distribution.

An analysis was conducted on baseline variables in the PCI and CABG groups with the aim of identifying those with a significant difference between the two groups. Subsequently, a univariate analysis was performed on those variables identified as different in both populations (age, sex, and diabetes mellitus). After confirming the statistical significance of these variables, the analysis was finalized using multiple logistic regression employing a “backward” strategy, considering the Z value associated with each variable (Wald test), estimated from the ratio of each coefficient to its standard error. This analysis included all variables that in the univariate analysis reached a significance level of 0.10 or less. 

Statistical significance was considered with a Type I error rate of less than or equal to 5% (p < 0.05; two-tailed). The IBM SPSS software version 25.0 was used for the study.

### Ethical aspects

This study was conducted in compliance with the National Law on Personal Data Protection No. 25326. The research was carried out following national ethical standards (City of Buenos Aires Law No. 3301, National Law on Clinical Research in Human Beings, Declaration of Helsinki, among others). Both registries were evaluated and approved by the ethics and research committees of the respective centers.

## Results

In the analysis, 1,848 patients with NSTE-ACS were included, of which 630 (34%) had unstable angina and 1,218 (66%) had non-ST-segment elevation MI. The median age was 54.8 (IQR: 53.7-56.0) years, and 24.2% were women. The median GRACE score was 112.4 (IQR: 110.8-113.9), and the median CRUSADE score was 23.9 (IQR: 23.2-24.6) **(**Table 1**)**. An initially invasive strategy was performed in 85.1% of patients during hospitalization, and 233 (12.6%) patients required CABG during the index hospitalization for NSTE-ACS. The median hospital stay was 3 days (IQR: 2-6), and the intrahospital mortality was 2.7% **(**Table 2**)**.

**Table 1:** Baseline characteristics of the population (n=1,848)

Variables	n (%)
Age (in years)*	54.8 (53.7-56.0)
Male gender	1,400 (75.8)
BMI*	28.4 (25.2-33.9)
Hypertension	1,354 (73.3)
Diabetes mellitus	504 (27.3)
Dyslipidemia	1,106 (59.9)
Current or past smoking	504 (27.3)
Previous bleeding	29 (1.6)
Previous acute coronary syndrome	560 (30.3)
Previous cardiac surgery	186 (10.1)
Previous stroke	113 (6.3)
LVEF*	42.1 (41.2-43.1)
GRACE score*	112.4 (110.8-113.9)
CRUSADE score*	23.9 (23.2-24.6)

* expressed as median and interquartile range.

BMI: body mass index expressed in kg/m2, LVEF: left ventricular ejection fraction, GRACE: The Global Registry of Acute Coronary Events, CRUSADE: Can Rapid Risk Stratification of Unstable Angina Patients Suppress Adverse Outcomes With Early Implementation of the ACC/AHA Guidelines.

**Table 2:** In-hospital management and evolution (n=1,848)

Variables	n (%)
Pre-treatment with P2Y12 inhibitors	915 (49.5)
Initial invasive strategy	1,573 (85.1)
Initial conservative strategy	275 (14.9)
CABG	233 (12.6)
Hospital stay (in days)*	3 (2-6)
Bleeding	
BARC 2	65 (3.5)
BARC 3	48 (2.6)
BARC 4	11 (0.6)
BARC 5	2 (0.1)
Ischemic events	152 (8.2)
Mortality	49 (2.7)

*expressed as median and interquartile range.

CABG: Coronary Artery Bypass Grafting, BARC: Bleeding Academic Research Consortium.

When comparing the groups that required CABG versus those who did not (i.e., those who underwent PCI or were not revascularized, the latter comprising only 2% of the patients), it was observed that those undergoing surgical revascularization were younger (51.5 vs. 55.7 years; p = 0.010), more frequently diabetic (38.2% vs. 25.7%; p = 0.001), had fewer previous CABG histories (11.2% vs. 2.1%; p = 0.011), and were more frequently male (90.1% vs. 73.7%; p = 0.001), with no significant differences in the rest of the observed clinical variables. Patients undergoing CABG had a hospital mortality of 6.8% (16 patients) compared to 2.0% (33 patients) in those who did not (p = 0.001); and a hospital stay time of 12 (IQR: 9-15.5) days versus 3 (IQR: 2-4) days (p = 0.001) **(**Table 3**)**.

**Table 3:** Analysis of variables according to the need for CABG (n=1,848)

Variables	Patients without CABG intervention (n=1,615)	Patients undergoing CABG (n=233)	P value*
Age (in years)**	55.7 (54.5-56.7)	51.5 (48.4-54.6)	0.010***
Male gender	1,190 (73.7)	209 (90.1)	<0.001
BMI**	28.4 (25.0-33.9)	28.7 (26.0-33.7)	0.930***
Hypertension	1,175 (72.8)	179 (76.8)	0.190
Diabetes mellitus	415 (25.7)	89 (38.2)	<0.001
Dyslipidemia	958 (59.3)	149 (63.9)	0.180
Current or past smoking	437 (27.1)	68 (29.2)	0.490
Previous bleeding	27 (1.7)	1 (0.4)	0.130
Previous acute coronary syndrome	490 (30.3)	70 (30.0)	0.920
Previous cardiac surgery	181 (11.2)	5 (2.1)	0.011
LVEF**	45 (18-55)	41.5 (32-55)	0.070***
GRACE score**	109 (89-133)	114 (95-129)	0.420***
CRUSADE score**	22 (14-33)	22 (14-31)	0.820***

* calculated by chi-square test..

** expressed as median and interquartile range.

*** calculated by the Mann-Whitney U test.

CABG: Coronary Artery Bypass Grafting, BMI: body mass index expressed in kg/m^2^, LVEF: left ventricular ejection fraction, GRACE: The Global Registry of Acute Coronary Events, CRUSADE: Can Rapid Risk Stratification of Unstable Angina Patients Suppress Adverse Outcomes With Early Implementation of the ACC/AHA Guidelines.

The multivariable analysis using logistic regression showed that the variables independently associated with CABG were: age (Odds Ratio [OR]: 0.99 per year, 95% confidence interval [CI]: 0.98-0.99; p=0.008), male sex (OR: 3.08, 95% CI: 1.87-5.10; p=0.001), history of previous CABG (OR: 0.14; 95% CI: 0.05-0.30; p=0.001), and diagnosis of diabetes (OR: 1.84, 95% CI: 1.31-2.57; p=0.001).

## Discussion

Our study demonstrated that 12.6% of patients with NSTE-ACS included in two multicenter registries required CABG during the index hospitalization, with certain baseline variables associated with this outcome.

Firstly, this proportion of 12.6% for CABG appears to be higher than that reported in other registries, where it ranges between 2% and 10% ^3^^,^^5^^-^^8^. n the GRACE registry, 10% of patients underwent surgery during hospitalization, based on data from patients included between 1999 and 2000 ^12^^,^^13^. Another registry from the United States showed a surgery rate of 11% between 2002 and 2008; however, 30% were performed during the index hospitalisation and the remaining 70% post-intervention ^14^. Many of these registries even demonstrate a reduction in the rate of patients undergoing surgery over time. For instance, the Israeli registry showed rates of 6.7% in 2000 and 1.7% in 2010 ^15^. This reduction in surgery rates appears to be related to advancements in coronary angioplasty techniques and the ability to address complex multivessel lesions with better long-term outcomes. The particular percentage of 12.6% in our registry may reflect the characteristics of several high-complexity centers that included a large proportion of patients in the registry, combined with patient-specific factors. 

Secondly, age was found to be a factor associated with undergoing CABG, with increasing age being inversely proportional to the likelihood of undergoing CABG. One of the first studies to attempt to associate clinical and angiographic variables to define which strategy to use was the SYNTAX 2 study published in 2013. This study evaluated seven clinical variables (age, renal function, COPD, female sex, presence of unprotected left main disease, peripheral vascular disease, and ventricular function) with a 5-year follow-up ^16^^,^^17^. In each of these variables, the Hazard Ratio for CABG vs. PCI was compared. It was observed that predictors favoring the CABG strategy with evidence of lower long-term total mortality were younger age, female sex, and reduced ejection fraction. Conversely, predictors favoring the PCI strategy were older age and COPD. It is likely that in our study, the older population is associated with a lower rate of CABG, secondary to the increased comorbidities, beyond the burden of atherosclerotic disease *per se*^18^. In the SYNTAXES substudy, in patients over 70 years old (31% of the total sample, with 575 patients), overall 10-year mortality was 44.1% in patients older than 70 years (mean: 75.8 ± SD: 3.6) vs. 16.6% in patients younger than 70 years (mean: 60 ± SD: 7.4) ^19^. The 5-year mortality, infarction or stroke (MACCE) rate was also higher in this subgroup of patients (35.1% vs. 23.0%). Thus, it seems reasonable for physicians to take age into account when making decisions. 

Thirdly, male gender increased the likelihood of undergoing CABG over threefold compared to receiving coronary angioplasty or medical treatment. This may be explained by the earlier progression of aterogénesis in males due to the absence of hormonal protection compared to females ^20^. In comparison to males, females usually exhibit a higher rate of risk factors but less extensive disease, less plaque rupture, smaller necrotic cores, with a similar plaque burden and smaller vessel caliber ^20^. 

Fourthly, diabetes showed an OR of 1.84 for CABG, which is consistent with the available evidence from the study in diabetic patients with multivessel disease (FREEDOM) published in 2012 ^21^. and a statistically significant difference in favor of CABG was found, resulting in a reduction in all-cause mortality and myocardial infarction at 5 years. Additionally, a higher rate of new revascularization was observed in the PCI group during the first 12 months of follow-up. Although there were no changes in the benefits of surgical revascularization when comparing patients with diabetes based on the need for insulin, overall mortality was higher in insulin users regardless of the strategy used ^22^^-^^25^. In the 5-year follow-up of the SYNTAX study, a higher rate of events was observed in the subgroup of diabetic patients compared to non-diabetic patients ^26^. However, a statistically significant difference was only observed when comparing the rate of new revascularization. 

Fifthly, 10% of the patients had a history of cardiac surgery, which appears to be proportionally high. Reoperation itself implies a significant increase in risk. Therefore, it seems sensible to attempt the resolution of these patients through an endovascular method.

As limitations of our study, we would like to highlight that the analysis presented earlier was conducted on an observational study, therefore, it is subject to biases to a greater extent than any experimental study, linked to data collection in an unaudited study, selected variables, etc. On the other hand, we had a relevant number of patients (n=1,848), although the myocardial revascularization group only comprised 12% of the total sample (n=233), compared to those who underwent other types of treatment (n=1,615). Finally, it should be noted that the analysis was performed based on a database obtained from patients hospitalized in high-complexity centers in ACBA and PBA, so the data obtained should not be generalized to any region of the country. 

In conclusion, in this analysis of two NSTE-ACS registries, the proportion of surgery during hospitalisation for the index event was 12.6%. Younger age, male sex, absence of previous surgery, and diagnosis of diabetes were independent predictors of the need for CABG during hospitalization.
